# Global health radiology planning using Geographic Information Systems to identify populations with decreased access to care

**DOI:** 10.7189/jogh.11.04073

**Published:** 2021-12-11

**Authors:** Rahul Sachdev, Shan Sivanushanthan, Natalie Ring, Anne-Marie Lugossy, Ryan W England

**Affiliations:** 1 The Johns Hopkins University School of Medicine, Baltimore, Maryland, USA; 2 Georgetown University School of Medicine, Washington, D.C., USA; 3 Russel H. Morgan Department of Radiology and Radiological Science, The Johns Hopkins Hospital, Baltimore, Maryland, USA; 4 RAD-AID International, Chevy Chase, Maryland, USA

## Abstract

**Background:**

Communities throughout northern Canada face significant health care disparities including decreased access to radiology. A medical hybrid airship is under development which aims to serve remote populations, requiring strategic outreach planning. This study aims to use geographic information systems (GIS) to identify (1) high risk and medically underserved patient populations in northern Canada and (2) potential landing sites for a medical airship to allow for mobile delivery of radiology services.

**Methods:**

The northern region of Canada extending from the Rocky Mountains to the Atlantic Ocean was analyzed using multi-variable, multi-weighted GIS modeling. Based on population distance from hospitals (50% weight), health centers (eg, clinic; 30% weight), remote communities (not connected to electric grid; 10% weight), and roads (10% weight), individuals were stratified into one of five health care accessibility index (HAI) categories (ranging from very low to very high severity). HAI (80% weight) was combined with population density (20%) to create a health care access severity index (HASI). Topographic and land cover data were used to identify suitable landing sites for the medical airship. A coordinate data set was made from georeferenced health care facilities, and infrastructure data was obtained from OpenStreetMap.

**Results:**

GIS analyzed 815 772 Canadians. Of this population, 522 094 (64%) were found to live ≥60 km from a hospital, 326 309 (40%) were ≥45 km from the nearest health center, 65 262 (8%) were within 30 km of a remote community, and 57 104 (7%) lived ≥1 km from the nearest road. Combined, the HASI identified 44% of the population as having decreased access to care (high or very high severity). Lastly, 27.5% of land analyzed was found to be suitable for airship operations.

**Conclusions:**

GIS identified medically underserved populations in northern Canada who may benefit from mobile radiology services. These techniques may help to guide future global health outreach efforts.

Access to radiology services is critical for disease prevention, surveillance, and treatment. Despite the importance of such services, residents of northern Canada, including the territories and the northern regions of the provinces, face significant health care disparities such as decreased health care utilization, lower life expectancy, and increased mortality. For instance, rates of both preventable and treatable mortality were found to be significantly higher for the northern territories compared to Canada as a whole [1]. Regarding life expectancy and infant mortality, residents in Nunavut and the surrounding circumpolar regions have greater infant mortality and shorter life expectancy when compared to the average Canadian [2,3]. While the etiology of these disparities is likely multifactorial, the geographical extent of the country exacerbates health conditions for many residents living in communities with limited access to hospitals and health care providers, including instrumental radiology services [4]. Identifying regions with limited access to health care, such as medical imaging, and developing interventions for increasing access are critical to improving health outcomes.

To reduce disparities in access to radiology services, geographic information systems (GIS) technology can be used for outreach planning and resource allocation. GIS is a computer mapping system that allows for the integration of multiple layers of data, including clinical and demographic information, to assess spatial relationships. Prior research has utilized GIS for disease surveillance, selective deployment of medical resources, and identifying medically underserved populations. For example, Rosero-Bixby et al used data derived from GIS to identify medically underserved Costa Rican populations based upon patient distance from hospital and clinics [5]. Researchers in Baltimore, Maryland, utilized GIS to identify regions with a high prevalence of gonorrhea in order to target future outreach efforts [6]. Daniels et al employed GIS in Alaska to identify regions with limited access to radiology services [7]. In turn, GIS has the potential to identify communities in northern Canada with limited access to health care and radiology.

Due to various infrastructure and climate constraints, regions in northern Canada with limited access to medical imaging may benefit from delivery of mobile radiology services using a medical hybrid airship (MHA). The MHA, developed in conjunction with RAD-AID International (Chevy Chase, Maryland, United States), Lockheed Martin (Bethesda, Maryland, United States), and Straightline Aviation (Bridgnorth, United Kingdom), allows for the deployment of mobile radiology services to regions with poor infrastructure. While traditional modes of transportation require a preexisting network of roads, airports, or railways, the MHA’s hybrid design allows access to nearly all regions with a relatively flat landscape and limited ground vegetation, including areas with snow and ice. Additionally, the MHA can carry 18 tonnes of equipment and 18 personnel (including outreach team members such as radiologists, technologists, and nurses), and is capable of operating in temperatures ranging from -40 to 49°C. These parameters allow for safe and effective operations in northern Canada and the surrounding circumpolar region [8,9]. The airship’s large payload capacity and vibration-free cargo bay are particularly useful for radiology outreach, as the majority of medical imaging equipment (such as x-ray, fluoroscopy, and CT scanners) is often both heavy and fragile. Additionally, the airship can be utilized to deploy customizable mobile health clinics specifically suited to addressing various health-related needs of a community [9]. At the time writing, the MHA’s Hybrid Type Design Criteria has preapproval of Federal Aviation Administration-type certification, and the airship prototype has been built and operated in test flights. For operational planning, GIS can be employed with the addition of topographic and land cover data to identify optimal landing sites for the MHA.

Addressing disparities in health care and radiology services for northern Canadian populations poses several unique challenges, emphasizing the need for careful outreach planning. The purpose of this study is to use GIS analysis to identify strategic regions and populations in northern Canada suitable for delivery of mobile radiology services using an MHA.

## METHODS

### Study site

The study site for GIS analysis was selected in the northern region of Canada based on potential bases of central operation for the MHA (to include Yellowknife, Northwest Territories and Schefferville, Quebec), range capabilities of the MHA, and geographic constraints. Based on these factors, the study site encompassed the northern region of Canada from the Rocky Mountains to the Atlantic Ocean, including the majority of Newfoundland and Labrador, Quebec, Nunavut, and the Northwest Territories; northern parts of Alberta, Saskatchewan, Manitoba, and Ontario; and northeast British Columbia.

### Data sources

All data used in this study were open source and freely available. Data used in GIS analysis (ArcMap 10.6, Redlands CA, USA) were pre-processed at a 90-m spatial resolution in projected coordinate system Canada Lambert Conformal Conic. Healthcare facility locations were collected from provincial government sites [10-12], and were georeferenced to create a point data set with coordinates for each facility. Two separate data sets were created from these locations, including 1) hospitals and 2) smaller facilities such as health centers and clinics.

Land cover and digital elevation model (DEM) data were obtained for determining suitable areas for MHA operations. Land cover data was collected at 30m resolution and was aggregated to 90m based on the maximum class value for each pixel. The data was classified to produce a map that included five categories including 1) dense vegetation, 2) medium vegetation, 3) sparse vegetation, 4) bare ground, and 5) urban areas. A DEM was created incorporating the Geobase DEM and Shuttle Radar Topography Mission elevation data. At a resolution of 90 m, this data was mosaicked and clipped to the study site.

Road location data was obtained from OpenStreetMap for use in the multicriteria evaluation (MCE) and accessibility analysis. Remote communities were identified from the Remote Communities Energy Database, which provided point locations of remote settlements not connected to an electric grid. This was used in the MCE to create a distance map from these unconnected locations.

### Multicriteria evaluation (MCEs)

MCEs were performed to create an index of populations stratified into five classes ranging from very low to very high severity, where areas of higher severity indicate populations with decreased access to health care and therefore most appropriate for allocating outreach services with the MHA.

An initial MCE was performed using distance from hospital locations (50% weight), health center locations (30% weight), roads (10% weight), and locations of remote communities not connected to the electric grid (10% weight) to create a health care accessibility index (HAI). These infrastructure variables were chosen as they are readily available, and because increased travel time is associated with decreased use of health care facilities [13-15]. Weights were based on their estimated impact on access to care, where the highest weight was given to distance to hospitals because these sites are most likely to have radiologic capabilities.

A second MCE was performed which combined the HAI (80% weight) with population density (20% weight) using WorldPop 2018 Census Data, where higher population was assigned higher severity, to create a final health care access severity index (HASI).

### Airship operating site criteria

Multiple variables were used to define acceptable zones for MHA operations (ie, takeoff and landing). GIS incorporated DEM and land cover data to identify terrain with less than a 3-degree slope, either low vegetation or bare ground, and encompassing a minimum area of 1200 × 3000 feet (366 × 914 m).

## RESULTS

### Healthcare Access Severity Index

**Figure 1:**
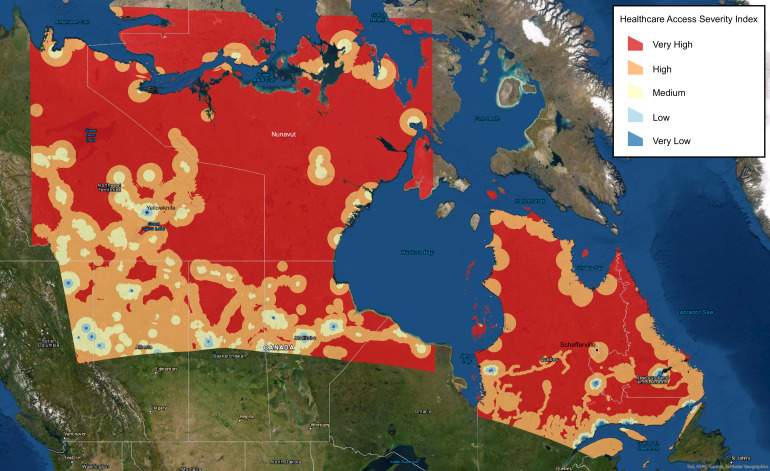
Geographic information systems (GIS) analysis mapping of the health care access severity index (HASI) in northern Canada, where higher severity areas indicate populations with decreased access to care.

GIS analyzed a total of 815 772 Canadians within the study site. Results of the multicriteria HAI evaluation with severity class distributions of health services, infrastructure, and roads are summarized in **Table 1**. Briefly, 522 094 (64%) Canadians were found to live ≥60 km from a hospital, with only 119 600 (15%) living within 10 km. 326 309 (40%) lived ≥45 km from the nearest health center (ie, clinic), and 79 082 (9.7%) lived between 30 to 45 km away from the nearest health center. While 57 104 (7%) lived ≥1 km from the nearest road, the vast majority (707 973; 87%) lived within 0.5 km of a road. 65 262 (8%) were found to live within 30 km of a remote community. Overall, GIS analysis identified 44% of the population as being underserved, as shown by a high or very high severity HASI score (**Table 2**). GIS mapping of northern Canada populations by HASI severity scores is shown in **Figure 1**.

**Table 1:** Geographic information systems (GIS) multicriteria evaluation of the health access index (HAI) for northern Canadians

Index variable	Severity class	Population (N)	Population (%)
**Distance from health center (km)**			
0 to 5	Very low	272 452	33.3%
5 to 15	Low	95 002	11.6%
15 to 30	Medium	44 555	5.46%
30 to 45	High	79 082	9.69%
45+	Very high	324 678	39.8%
**Distance from hospitals (km)**			
0 to 10	Very low	119 600	14.7%
10 to 20	Low	33 819	4.15%
20 to 40	Medium	57 083	7.00%
40 to 60	High	82 876	10.2%
60+	Very high	522 389	64.0%
**Distance from roads (km)**			
0 to 0.5	Very low	707 973	86.8%
0.5 to 1	Low	53 985	6.62%
1 to 2	Medium	28 137	3.45%
2 to 20	High	21 904	2.69%
20+	Very high	3768	0.46%
**Distance from remote communities (km)**			
40+	Very low	747 873	91.7%
30 to 40	Low	2788	0.34%
20 to 30	Medium	5463	0.67%
10 to 20	High	16 484	2.02%
0 to 10	Very high	43 159	5.29%

**Table 2:** Healthcare Access Severity Index (HASI) distribution for northern Canadians

MCE Severity Index	Population (N = 815 768)	Population (%)
Very low	130 012	16%
Low	57 951	7.1%
Medium	270 612	33.2%
High	335 166	41.1%
Very high	22 027	2.7%

MCE – multicriteria evaluation

### Airship operations

**Figure 2:**
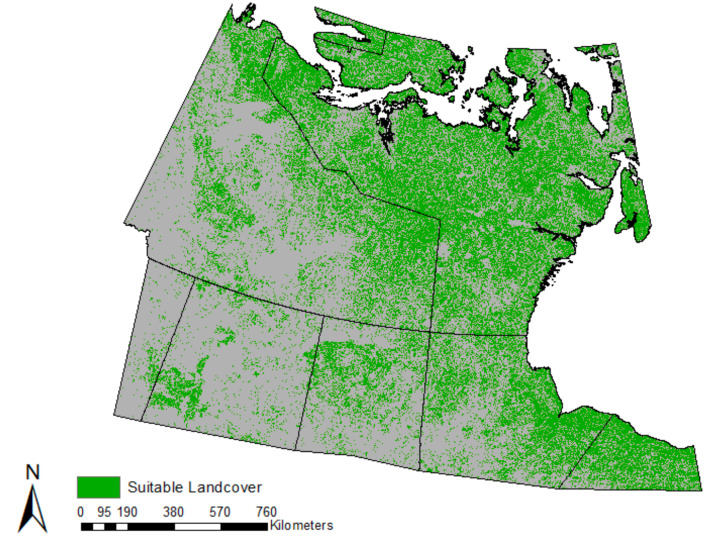
Geographic information systems (GIS) analysis mapping of suitable medical hybrid airship landing areas (green) in northern Canada, determined from required land slope and ground vegetation constraints.

Based on GIS analysis, 27.5% of land analyzed within the study site was found to be suitable for MHA operations when accounting for required land slope and ground vegetation. GIS mapping produced a visual illustration of suitable landing areas for the airship for comparison to HASI outcomes, and strategic planning purposes (**Figure 2**).

## DISCUSSION

This study utilized GIS to identify underserved areas of northern Canada for radiology outreach planning. By employing an MCE, this study revealed that 44% of the population was identified as having decreased access to health care, with either a high or very high HASI score. This finding supports previous studies which have also noted differences in access to care for Northern Canadians when compared to the rest of the country [15,16-18]. For example, the metric of distance to nearest physician has been used to describe the differences in health care access by territory [16]. However, to our knowledge, this is the first study to use a multicriteria, weighted-variable analysis to identify high-risk populations in this region.

The results of this analysis support the urgent need to provide medical services to the underserved communities of northern Canada. North of the densely inhabited US-Canadian border corridor, health care and radiology services are not proportionate to the population, with only 13.6% of the country’s family physicians, less than 3% of specialists, and no radiologists working in rural or remote areas of northern Canada [4,17]. Currently, patients in northern Canada are often medically transported long distances to urban areas in order to provide necessary medical care, which is associated with a significant cost burden. For instance, 4%-5% of the Northwest Territory’s Department of Health and Social Services and 18%-20% of Nunavut’s Department of Health’s budget has been needed for medical travel [18]. The number of residents requiring these services, as well as the complexity and cost of specialized services needed, continues to increase [17,19]. Furthermore, remote indigenous populations avoid travelling outside of the community for non-emergent medical services [20]. With the development of MHAs, this technology may provide a sustainable pathway to providing radiology and medical services in a cost-effective manner, enabling timely diagnosis and treatment of illnesses, and decreasing the need for patients to leave their communities in order to seek preventative, diagnostic, or therapeutic care.

GIS identified 27.5% of analyzed sites as suitable for MHA operations, highlighting the geographical and topographical complexity of northern Canada. Delivering radiological services to this at-risk population poses logistical challenges, and operational values (base of operations, loading and unloading, payload capacity, etc.) must be accounted for. This study demonstrated the diverse capabilities of GIS analysis, including the production of a HASI and identification of possible airship landing sites, together allowing for improved global health outreach planning.

While this report offers one potential tool for helping reduce health disparities, namely by addressing structural barriers through local, mobile radiology services, the current health care disparities facing northern Canadians are a byproduct of multiple factors which must be addressed in tandem. For example, indigenous populations, who speak mainly Cree, Inuktitut, or Ojibway, have limited access to translators or appropriately translated materials [20,21]. Culturally, the Canadian health care system frequently fails to incorporate or acknowledge traditional indigenous practices, such as smudging, which involves burning dried plants [22]. Incorporating these traditional practices may allow for greater engagement and trust. Future interventions to address health care disparities must be cognizant of these factors to achieve successful outcomes.

This study’s findings are consistent with previous research on GIS utilization in health care outreach in a variety of settings. GIS has previously been used to demonstrate relationships between geography and public health measures like lead poisoning, smoking, and obesity prevalence [23,24]. In radiology, this analytic method has been used to reveal disparities in access to computed tomography (CT) facilities in less densely populated areas [25,26]. Additionally, a study of populations in Alaska, whose decreased access to health care when compared with the contiguous US mirrors that of northern to southern Canadians, demonstrated similar utility of GIS in global health outreach planning and resource allocation [7]. Our analysis builds on this body of work, and further demonstrates the wide geographical differences in access to care. In addition, it also highlights the wide customizability of multicriteria, weighted-variable GIS analysis to various geographically complex terrains, and provides a useful blueprint for future global health outreach projects using freely available and open-source data.

There are limitations to this study. Hospital infrastructure and employment data are continuously evolving, and data from this study used to identify areas with limited or no access to radiological services for outreach planning may be outdated once projects are executed. However, the reproducibility of these GIS analytic techniques with up-to-date open-source data helps to address this limitation. Additionally, the MCE also used distance from health facilities as a marker for a population’s access to radiological services; however, the presence and functional status of various radiological services provided at each facility were not known. Individualized analysis and detailed knowledge of each hospital and health center in the study area would be required to truly capture the severity of decreased access to radiological services. Lastly, while this study provides a theoretical framework for planning mobile health outreach, practical implementation of airship-based radiologic services would require further research and planning based on the Canadian national health system, a more granular understanding of health-related needs of each community, and various logistical challenges unique to each community being served.

## CONCLUSION

Many residents of northern Canada, including a large proportion of indigenous communities, face limited access to medical and radiology services critical for disease prevention and management, and are often dependent on medical evacuation for care in the acute setting. Using a multicriteria GIS approach based on critical infrastructure metrics, this study identified medically underserved populations and land suitable for medical hybrid airship operations, enabling strategic global health outreach planning. Improving local access to radiology services for these at-risk communities is critical for reducing health disparities, and this novel application of GIS may help bridge such disparities.
